# Identification of crucial genes through WGCNA in the progression of gastric cancer

**DOI:** 10.7150/jca.95757

**Published:** 2024-04-23

**Authors:** Rui Liu, Jie Liu, Qiang Cao, Yanpeng Chu, Hao Chi, Jun Zhang, Jiangping Fu, Tianchi Zhang, Linguang Fan, Chaozhong Liang, Xiufang Luo, Xiaoli Yang, Bo Li

**Affiliations:** 1 Vascular surgery Department, The Affiliated Hospital of Southwest Medical University, Lu Zhou, China.; 2 Department of gastrointestinal surgery, Meishan People 's Hospital, Meishan, China.; 3 Department of General Surgery (Hepatopancreatobiliary Surgery), The Affiliated Hospital of Southwest Medical University, Sichuan, China.; 4 Academician (Expert) Workstation of Sichuan Province, Metabolic Hepatobiliary and Pancreatic Diseases Key Laboratory of Luzhou City, The Affiliated Hospital of Southwest Medical University, Sichuan, China.; 5 Department of general surgery, Dazhou Central Hospital, Dazhou, China.; 6 School of Medicine, Macau University of Science and Technology, 999078, Macau, China.; 7 Medical College, Sichuan University of Arts and Science, Dazhou, China.; 8 Oncology department, Dazhou Central Hospital, Dazhou, China.; 9 Geriatric department, Dazhou Central Hospital, Dazhou, China.

**Keywords:** Gastric cancer, Tumor progression, WGCNA, Hub gene, Immunohistochemistry

## Abstract

**Background:** To explore the hub gene closely related to the progression of gastric cancer (GC), so as to provide a theoretical basis for revealing the therapeutic mechanism of GC.

**Methods:** The gene expression profile and clinical data of GSE15459 in Gene Expression Omnibus (GEO) database were downloaded. The weighted gene co-expression network analysis (WGCNA) was used to screen the key modules related to GC progression. Survival analysis was used to assess the influence of hub genes on patients' outcomes. CIBERSORT analysis was used to predict the tissue infiltrating immune cells in patients. Immunohistochemical staining was conducted to further verify the expression of hub genes.

**Results:** Through WGCNA, a total of 26 co-expression modules were constructed, in which salmon module and royalblue module had strong correlation with GC progression. The results of enrichment analysis showed that genes in the two modules were mainly involved in toll-like receptor signaling pathway, cholesterol metabolism and neuroactive ligand-receptor interaction. Six hub genes (*C1QA*, *C1QB*, *C1QC*, *FCER1G*, *FPR3* and *TYROBP*) related to GC progression were screened. Survival analysis showed overall survival in the high expression group was significantly lower than that in the low expression group. CIBERSORT analysis revealed that immune characteristics difference between patients in early stage and advanced stage. Immunohistochemical results confirmed that *C1QB*, *FCER1G*, *FPR3* and *TYROBP* were significantly associated with disease progression in GC.

**Conclusion:** Our study identified that *C1QB*, *FCER1G*, *FPR3* and *TYROBP* played important roles in the progression of GC, and their specific mechanisms are worth further study.

## 1. Introduction

Gastric cancer (GC) is the fifth most common cancer in the world, with more than one million new cases occurring every year. The early symptoms of GC are not obvious, and about 50% of GC patients are already in the advanced stage when they are found, thus losing the best opportunity for surgical treatment. As GC is often diagnosed at an advanced stage, it has a high mortality rate, making it the third leading cause of cancer-related deaths, with 768,793 deaths worldwide in 2020 1. However, 25-40% of patients with treated GC will have recurrence and metastasis due to tumor heterogeneity 2, which is a major challenge in the treatment of GC.

GC is a heterogeneous disease with high histological and molecular diversity and a complex genomic landscape, thus challenging the definition of clinical and therapeutic strategies 2. To date, there is no gold standard therapy for GC, and the treatment options are mainly based on the stage of disease and/or the presence of biomarkers. For the early-stage disease, surgery is the only curative approach in the treatment of GC. A large-scale study has demonstrated that the addition of chemotherapy in stage II and III patients can add a survival benefit compared to surgery alone 3. Targeted therapies for GC are anti-human epidermal growth factor receptor 2 (HER2) and anti-vascular therapy 4. Noteworthily, due to the known heterogeneity of GC cases, only specific subpopulations of GC would benefit from immune therapy 5. Although various treatments such as radiotherapy and chemotherapy, neoadjuvant chemotherapy and immunotherapy can prolong the survival of patients to a certain extent, it is still difficult to significantly improve the overall survival rate of patients with advanced GC 6, 7. Therefore, in-depth exploration of the molecular mechanism of GC metastasis and the search for molecular targets in the process of cancer metastasis and invasion are of significant clinical significance for the control of GC metastasis and the development of targeted therapeutic drugs.

Although many excellent algorithms have been proposed for the study of important molecules 8, 9, in the current study, we conducted a comprehensive bioinformatics analysis between early-stage GC tissues and advanced stage GC tissues via weighted gene co-expression network analysis (WGCNA) 10, 11. And hub genes identification, biological processes and tumor immune status were determined based on comprehensive bioinformatics analyses. The relationship between hub genes and patient outcomes was also investigated. Finally, we validated the expression of the hub gene in our cohort by immunohistochemistry (IHC).

## 2. Materials and Methods

### 2.1 Data acquisition

The GC-related expression profiling by array data (GSE15459) and clinical information were downloaded from the Gene Expression Omnibus (GEO) database (https://www.ncbi.nlm.nih.gov/geo/). The data set included 192 primary tumor tissues from GC patients with an average age of 64.37 (23.4-92.4) years, and 125 patients were male and 67 patients were female. Among them, 31 patients were in stage 1, 29 patients were in stage 2, 72 patients were in stage 3 and 60 patients were in stage 4 at the time of surgery, respectively. The annotation information of GSE15459 was obtained through GPL570 [HG-U133_Plus_2] Affymetrix Human Genome U133 Plus 2.0 Array. After obtaining the gene expression matrix of GSE15459, the gene without gene name was deleted by comparing with the micro-array annotation information table. For the repeated genes in the matrix, the genes with the largest total sample mean were retained for subsequent analysis.

### 2.2 Weighted gene co-expression network analysis (WGCNA)

The “WGCNA” package of R (version: 3.6.3) was used to construct the co-expression network. Hierarchical clustering analysis was performed in the gene expression data of 192 GC samples to identify outliers. The R function “pickSoftThreshold” was applied to screen the appropriate soft threshold power β for standard scale free network establishment. In this study, the soft threshold was set to 10. The weighted adjacency matrix is transformed into a topological overlap metric matrix (TOM) to estimate its connectivity in the network. The clustering tree of TOM matrix was constructed by using the average link hierarchical clustering method. In this study, the minimum gene module size was set to 30 to obtain the appropriate module, and the threshold for merging similar modules was set to 0.25. The gene significance (GS) and module membership (MM) were calculated to relate modules to clinical traits. For the identified modules, the important modules that need further analysis were determined according to their correlation coefficients r and P values.

### 2.3 Gene Ontology (GO) and Kyoto Encyclopedia of Genes and Genomes (KEGG) pathways enrichment analysis

GO functional enrichment analysis and KEGG enrichment analysis were performed on the genes in the key modules related to GC progression using online STRING (https://string-db.org/). The false discovery rate ≤ 0.05 indicated that the enrichment results were statistically significant. The results of GO analysis mainly studied the related pathways of enrichment of these genes in biological processes, molecular functions and cellular components. The results of GO and KEGG were visualized by using the “ggplot2” package of R software. The results of top 10 in each GO terms of GO analysis were presented in the form of bar chart, and the enrichment results of KEGG were presented in the form of bubble chart.

### 2.4 Hub gene identification and survival analysis

The GS and MM values in the key gene module of GC were calculated respectively, and the key genes in the key module were screened according to the screening criteria recommended by the WGCNA official website (|MM| > 0.8 & |GS| > 0.2). The selected key genes were further subjected to expression analysis in the GEPIA (http://gepia.cancer-pku.cn/; Match TCGA normal and GTEx data) database to find genes significantly related to different stages of GC patients for subsequent verification. Meanwhile, Kaplan-Meier survival analysis followed by log-rank test was performed to evaluate the impact of hub genes on the prognosis of patients.

### 2.5 Gene Set Enrichment Analysis (GSEA) of hub genes

To study the potential function of hub genes, the “clusterProfiler” package of R was applied to perform GSEA analysis. The samples were divided into two groups according to the median expression of hub genes, and the h.all.v7.4.entrez.gmt was chosen for analysis. The “ggplot2” package was used to visualize the significant functional pathways involved in hub genes. And specific pathways were visualized with “enrichplot” package.

### 2.6 Evaluation of tissue infiltrating immune cells

The CIBERSORT deconvolution algorithm was employed to predict the immune cell fractions in GSE15459 datasets based on gene expression profiles, according to the known reference set LM22 (leukocyte signature matrix) 12. The relationship of hub genes and tissue infiltrating immune cells were assessed via the method of Spearman analysis.

### 2.7 Immunohistochemical validation of hub genes

In this study, 20 patients with GC who were surgically removed between March 2022 and May 2022 from Dazhou Central Hospital were collected in accordance with the policy of the Ethics Committee of the Dazhou Central Hospital (No. 2022032). According to the tumor-node-metastasis (TNM) cancer staging system of the American Joint Committee on Cancer (Version 8), 10 patients were in early stage (with a mean age of 68.3; 60% were male) and 10 patients were in advanced stage (with a mean age of 68.7; 50% were male). The detailed demographic information of the patients is provided in Supplementary File 1. Tumor tissues were fixed with 4% paraformaldehyde, embedded in paraffin and sliced into 4 μm-thick sections following a standard protocol. *C1QA* monoclonal antibody (1:100, Cat.67063-1-Ig, Proteintech), *C1QB* rabbit pAb (1:100, Cat.382301, ZENBI0), *C1QC* polyclonal antibody (1:100, Cat.16889-1-AP, Proteintech), Anti-*FPR3* (1:100, Cat.ab188785, abcam), *DAP12* (*TYROBP*) rabbit pAb (1:100, Cat.383147, ZENBI0) and *FCER1G* (1:100, Cat.CQA4448, Cohesion) were used as primary antibodies, and working solution form Dako REAL™ EnVision™ test (Cat.K5007) were as secondary antibody. The tissue samples of two patients had been detached in the experiment, and the final experimental results could not be obtained. Therefore, only 9 patients in each group were included in the final immunohistochemical analysis. The results of the statistical analysis of IHC were performed by Aperio ImageScope software (Vista, CA, USA) and the difference was compared based on H-score.

## 3. Results

### 3.1 Construction of weighted gene co-expression network and key gene modules identification

**Figure 1:**
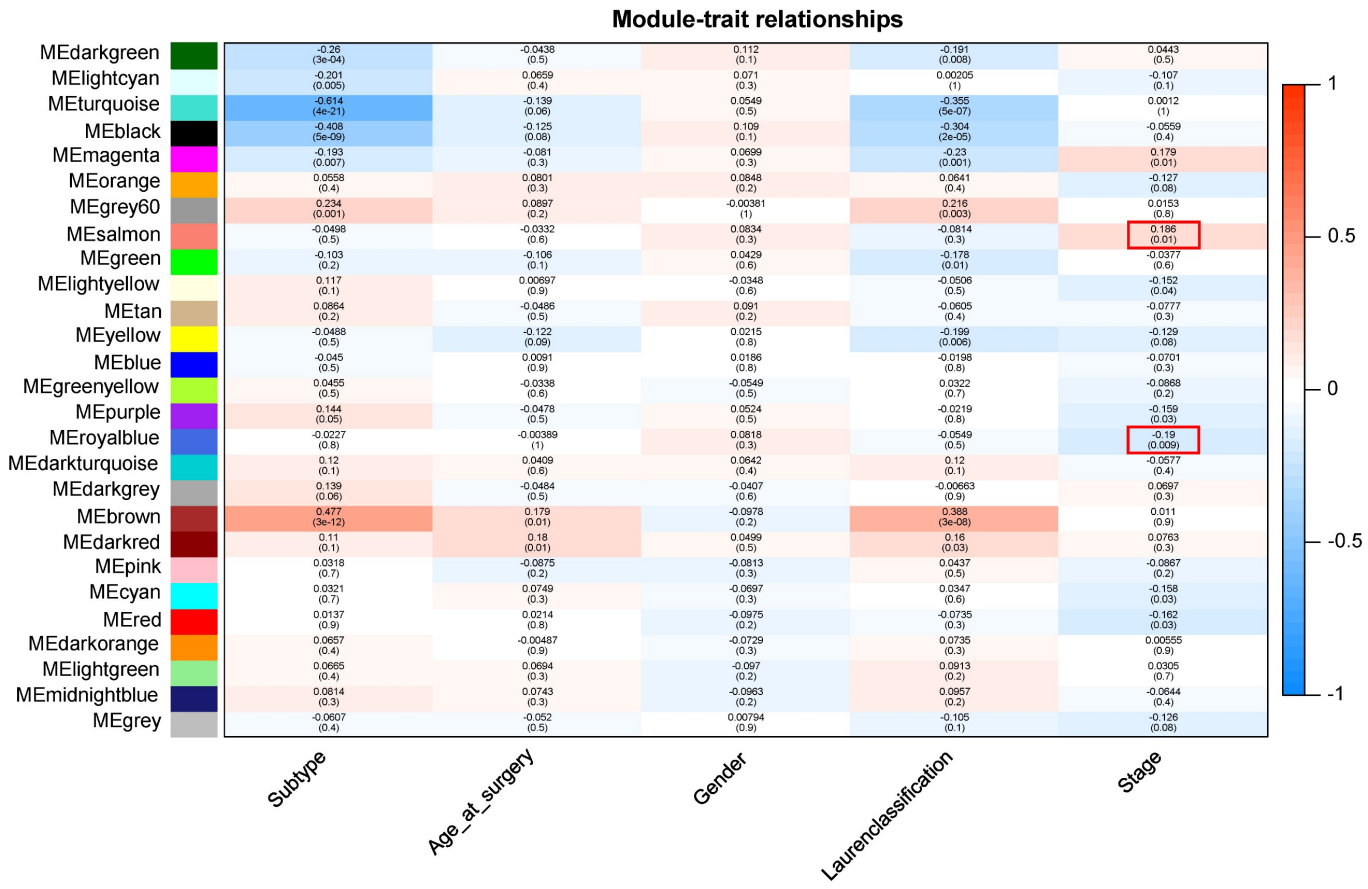
Module-trait relationship. Each row indicates a module eigengene and each column indicates a trait.

Patients with different stages have significantly different prognosis, and in general, the earlier the stage (Early stage included stage 1 and stage 2 patients, advanced stage included stage 3 and stage 4 patients), the better the prognosis (**Supplementary Figure 1**). After preprocessing GSE15459 data, the expression matrix containing 21,653 genes was finally obtained for analysis. The average clustering method was used to cluster 192 samples. The height of clustering was set to 5e+5 (marked by red line) (**Supplementary Figure 2A**), and the abnormal sample GSM387791 was deleted according to the clustering tree. After removing the abnormal sample, sample clustering containing corresponding sample information was obtained for subsequent WGCNA analysis (**Supplementary Figure 2B**). Through the establishment of a scale-free network, gene modules highly related to disease stage were screened, and the soft threshold was selected as 10 (R^2^ = 0.95), and the modules were finally determined (non-clustering modules were gray) (**Supplementary Figure 3**). Finally, 26 gene modules were identified, among which 7 modules were significantly correlated with tumor stage in GC. ME-salmon (r=0.186, *P*=0.01) and ME-royalblue (r=-0.19, *P*=0.009) were selected as the modules with the largest correlation coefficient with tumor stage, which contained 164 genes (**Figure 1**).

### 3.2 Gene function and pathway enrichment analysis

**Figure 2:**
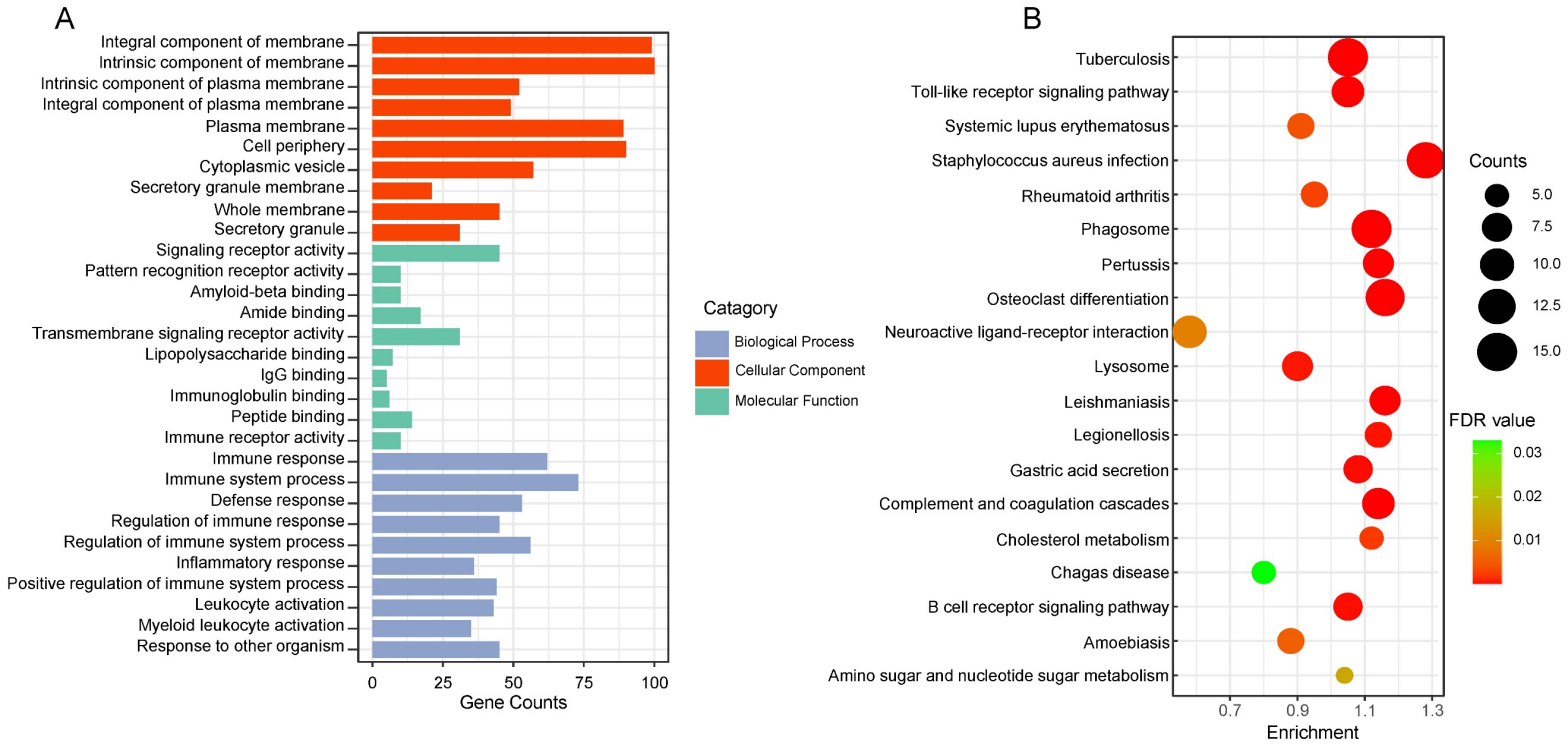
The functional enrichment analysis of gene in the two clinically important modules. (A) Top 10 GO terms in each category of GO enrichment analysis. (B) Significantly enriched KEGG pathways. FDR: false discovery rate.

GO and KEGG enrichment analysis of 164 genes in salmon and royalblue modules to gain insight into biological significance of these genes. The results showed that GO functional annotation significantly enriched 360 biological process items, 47 cellular component items and 21 molecular function items (Detailed GO terms were in supplementary file 2). All significant molecular functions, cellular component, and top 10 biological processes are shown in bar charts in **Figure 2A**, mainly involved in Integral component of membrane, Signaling receptor activity, Immune response. Genes in the KEGG pathway enrichment analysis module were significantly enriched in 19 pathways, including Toll-like receptor signaling pathway, Neuroactive ligand-receptor interaction, Lysosome, Gastric acid secretion, Cholesterol metabolism (**Figure 2B**).

### 3.3 Identification and function analysis of hub genes

**Figure 3:**
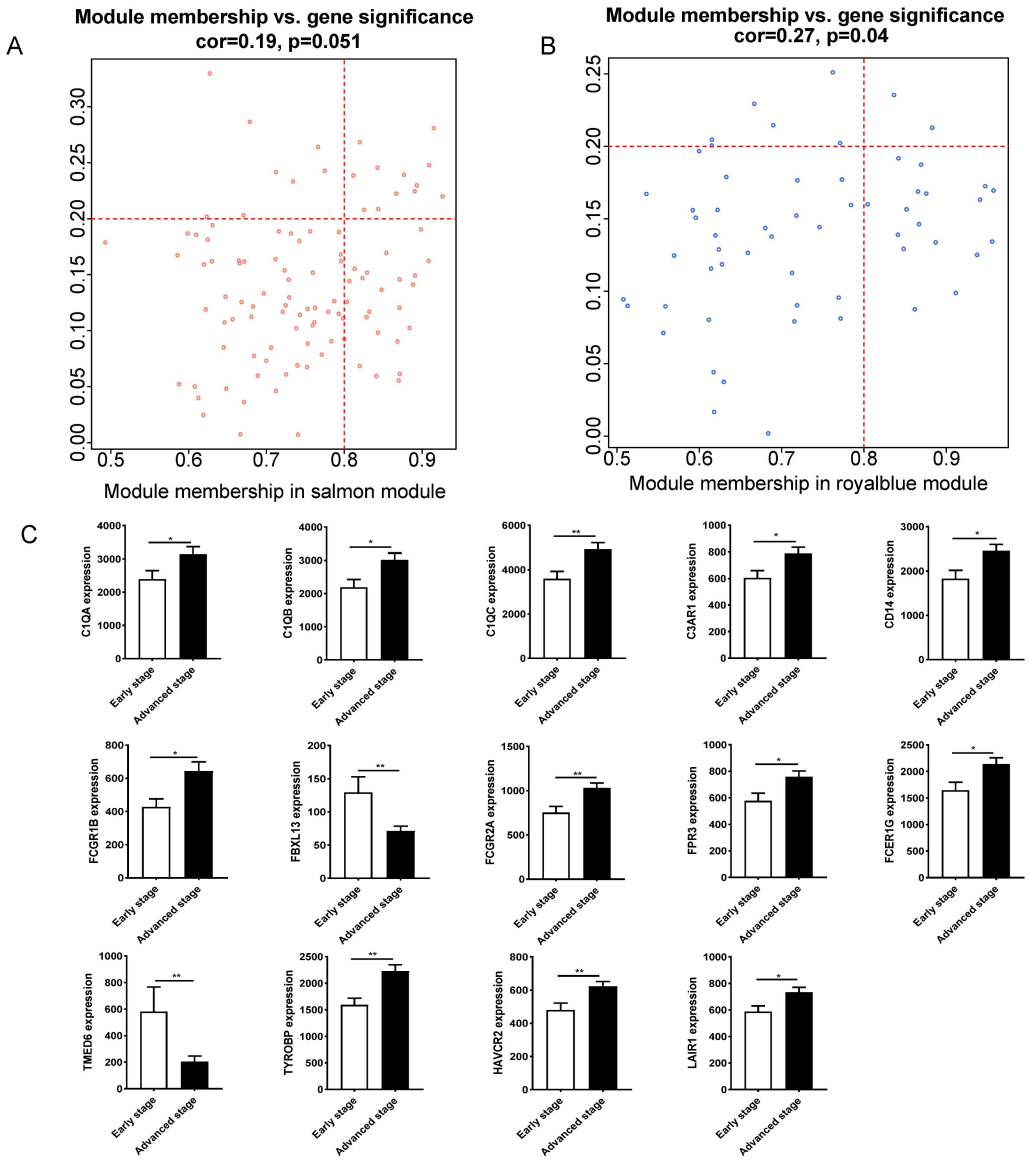
Hub gene identification between early stage and advanced stage of gastric cancer. (A)The scatterplot describing the relationship between MM and GS in salmon module. (B) The scatterplot describing the relationship between MM and GS in the royalblue module. (C) Fourteen hub genes expression between early stage and advanced stage of gastric cancer in GSE15459 cohort.

**Figure 4:**
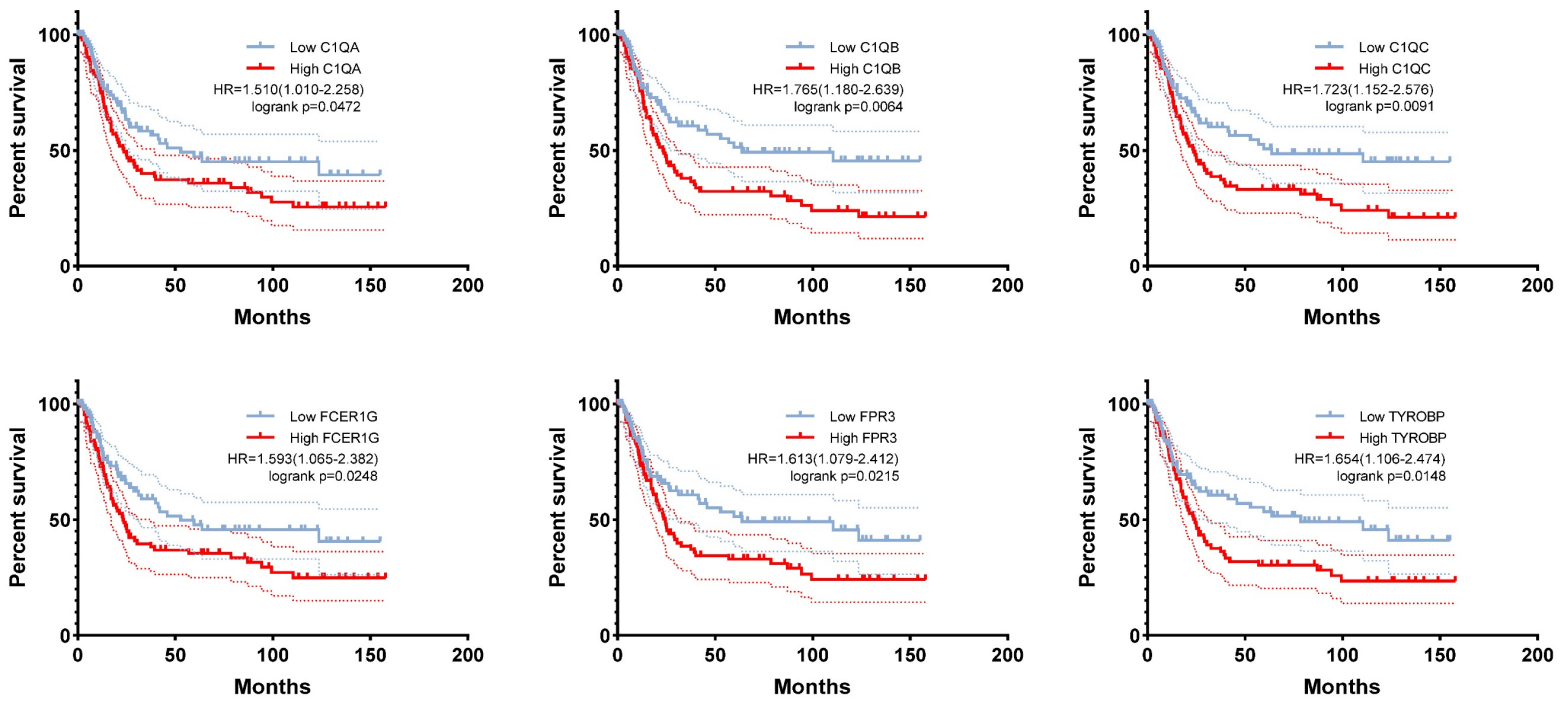
The correlation between *C1QA*, *C1QB*, *C1QC*, *FCER1G*, *FPR3*, *TYROBP* expression and the prognosis of gastric cancer was analyzed using GSE15459 cohort.

**Figure 5:**
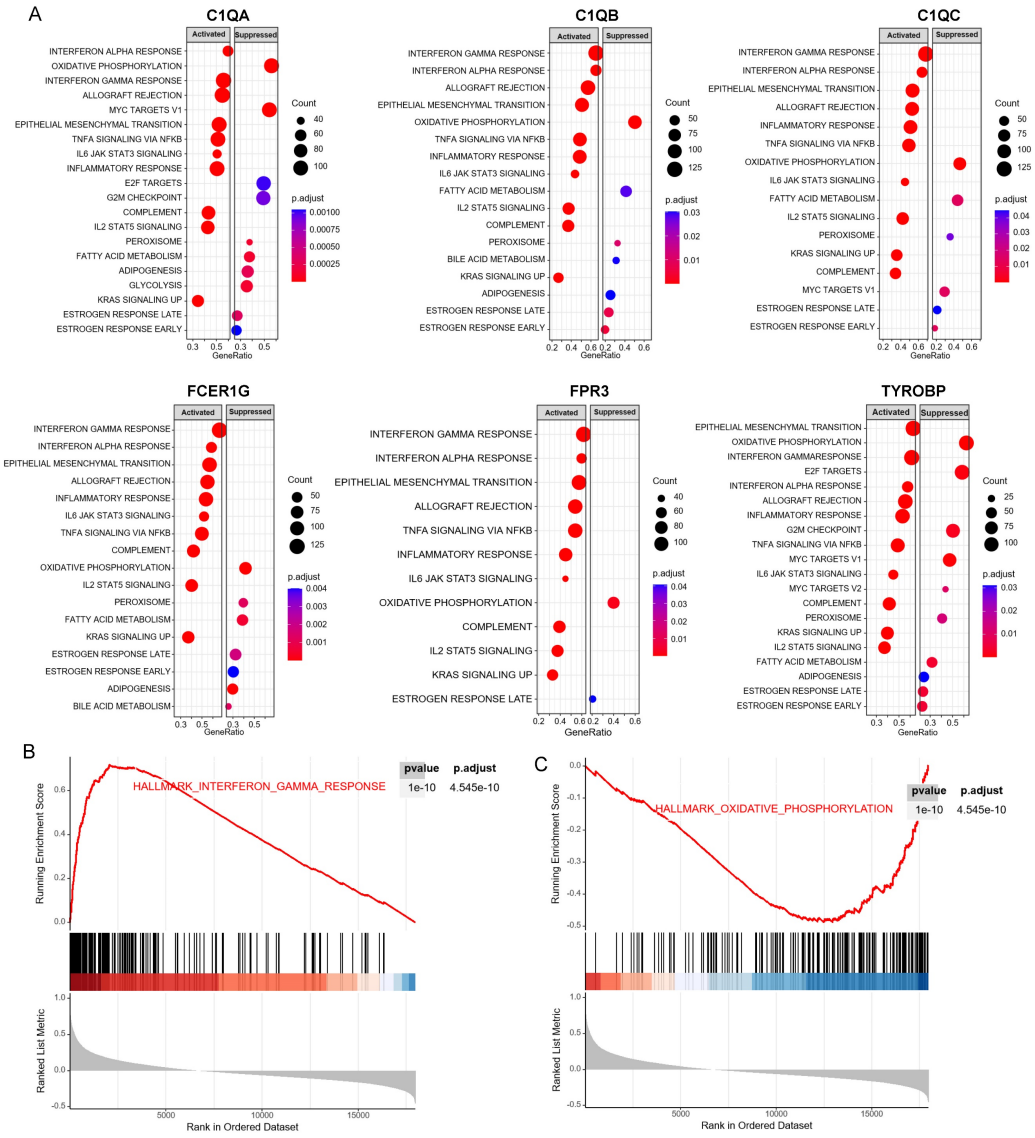
GSEA for samples with high hub gene expression and low hub gene expression. (A) The enriched gene sets in HALLMARK collection by samples of high *C1QA*, *C1QB*, *C1QC*, *FCER1G*, *FPR3*, *TYROBP* expression, respectively. (B) Gene set enriched in the interferon gamma response (*p*.adjust = 4.545e-10, NES =3.33, *p*-value = 1e-10). (C) Gene set enriched in the oxidative phosphorylation (*p*.adjust = 4.545e-10, NES =-2.39, *p*-value = 1e-10). NES: normalized enrichment score. GSEA: Gene Set Enrichment Analysis.

Fourteen hub genes were identified from the salmon and royalblue modules by setting the screening criteria of |MM| > 0.8 and |GS|> 0.2 (**Figure 3A-B**). The difference analysis results further demonstrated that *C1QA*, *C1QB*, *C1QC*, *C3AR1*, *CD14*, *FCER1G*, *FCGR1B*, *FCGR2A*, *FPR3*, *HAVCR2*, *LAIR1* and *TYROBP* were significantly over-expressed in advanced patients, while *FBXL13* and *TMED6* were significantly over-expressed in early patients (**Figure 3C**). The GEPIA database was further used to analyze these genes and it was found that 12 genes except for *FCGR1B* and *FBXL13* were significantly different between GC tissues and normal tissues (*P*<0.05). Among the 12 genes, *C1QA*, *C1QB*, *C1QC*, *FCER1G*, *FPR3*, *HAVCR2*, *LAIR1* and *TYROBP* displayed obvious differences in different stages (*P*<0.05) (**Supplementary Figure 4**). According to the median expression of genes, those below this value are low-expression groups, and those above this value are high-expression groups. Kaplan-Meier survival analysis in GSE15459 cohort further indicated that the *C1QA* (HR=1.510, *P*=0.0472), *C1QB* (HR=1.765, *P*=0.0064), *C1QC* (HR=1.723, *P*=0.0091), *FCER1G* (HR=1.593, *P*=0.0248), *FPR3* (HR=1.613, *P*=0.0215) and *TYROBP* (HR=1.654, *P*=0.0148) were significantly associated with overall survival of GC (**Figure 4**, **Supplementary Figure 5**). GSEA was performed to identify the functional enrichment of the above high expression genes and above low expression genes respectively (**Figure 5**). HALLMARK enrichment term exhibited that high expression of *C1QA* was mainly associated with interferon alpha response activation and oxidative phosphorylation suppression, high expression of *C1QB*, *C1QC*, *FCER1G* and *FPR3* were all mainly associated with interferon gamma (INFγ) response activation and oxidative phosphorylation suppression, and high expression of *TYROBP* was mainly associated with epithelial mesenchymal transition activation and oxidative phosphorylation suppression (**Figure 5A**). These results suggested that these genes played a major role in INFγ response activation (**Figure 5B**) and oxidative phosphorylation suppression (**Figure 5C**).

### 3.4 Differences in immune characteristics between early and advanced stage

**Figure 6:**
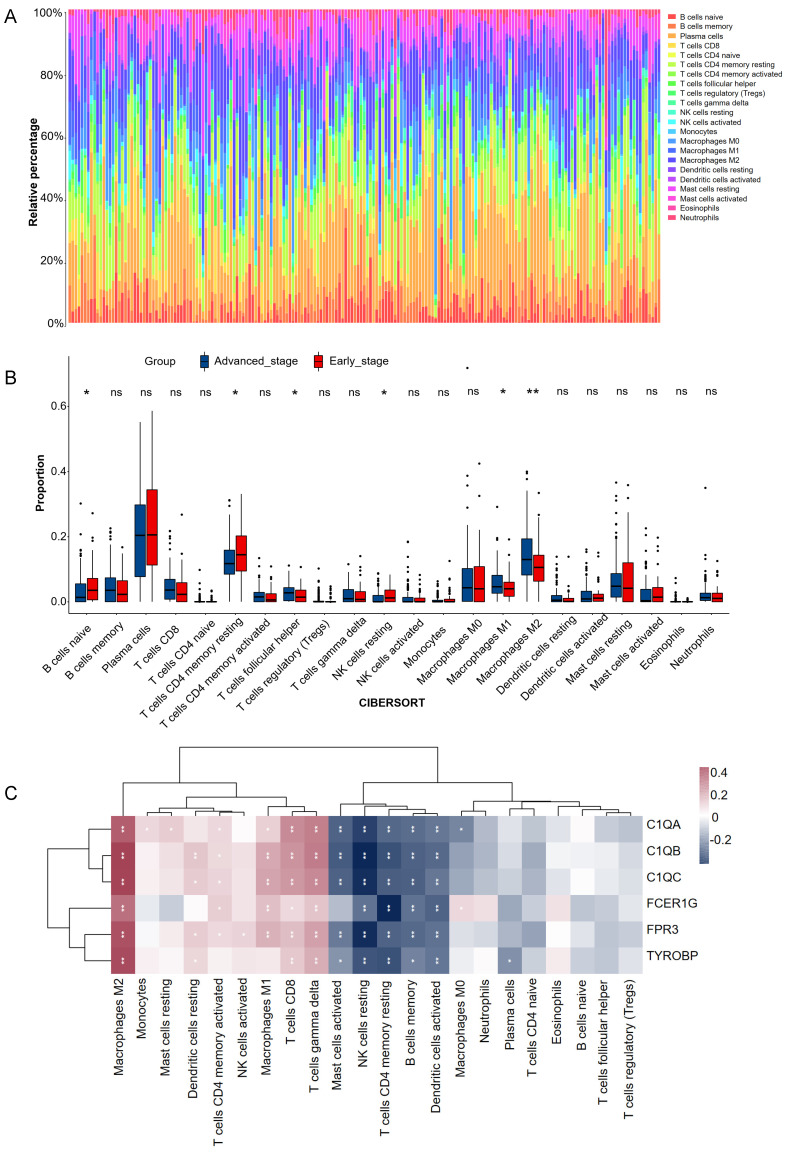
Estimation of tissues infiltrating immune cell types between early stage and advanced stage patients in the GSE15459 cohorts via CIBERSORT. (A) Stacked barplots show the relative composition of 22 immune cell subsets in 191 gastric cancer patients. (B) The boxplots show tissues infiltrating immune cell difference between early stage and advanced stage gastric cancer patients. Data were assessed via the method of wilcox test. * *p*-value < 0.05, ** p-value < 0.01, ns, no significance. (C) Heatmap of 6 hub genes and tissues infiltrating immune cell. Data were assessed via the method of Spearman analysis. * *p*-value < 0.05, ** *p*-value < 0.01.

CIBERSORT analysis revealed that patients in early stage had significantly lower levels of T cells follicular helper, macrophages M1, and macrophages M2 than those in advanced stage. However, the levels of B cell naive, T cells CD4 memory resting, and NK cells resting were significantly higher than those in advanced stage (**Figure 6A-B**). The correlations between the 6 hub genes and immune cell types were also calculated for all patients (**Figure 6C**). Among the significant difference immune cell types, all six hub genes were significantly positive with macrophages M2, significantly negative with T cells CD4 memory resting, and NK cells resting.* C1QA*, *C1QB*, *C1QC*, *FCER1G* and *FPR3* were significantly positive with macrophages M1.

### 3.5 Immunohistochemistry validation of hub genes

**Figure 7:**
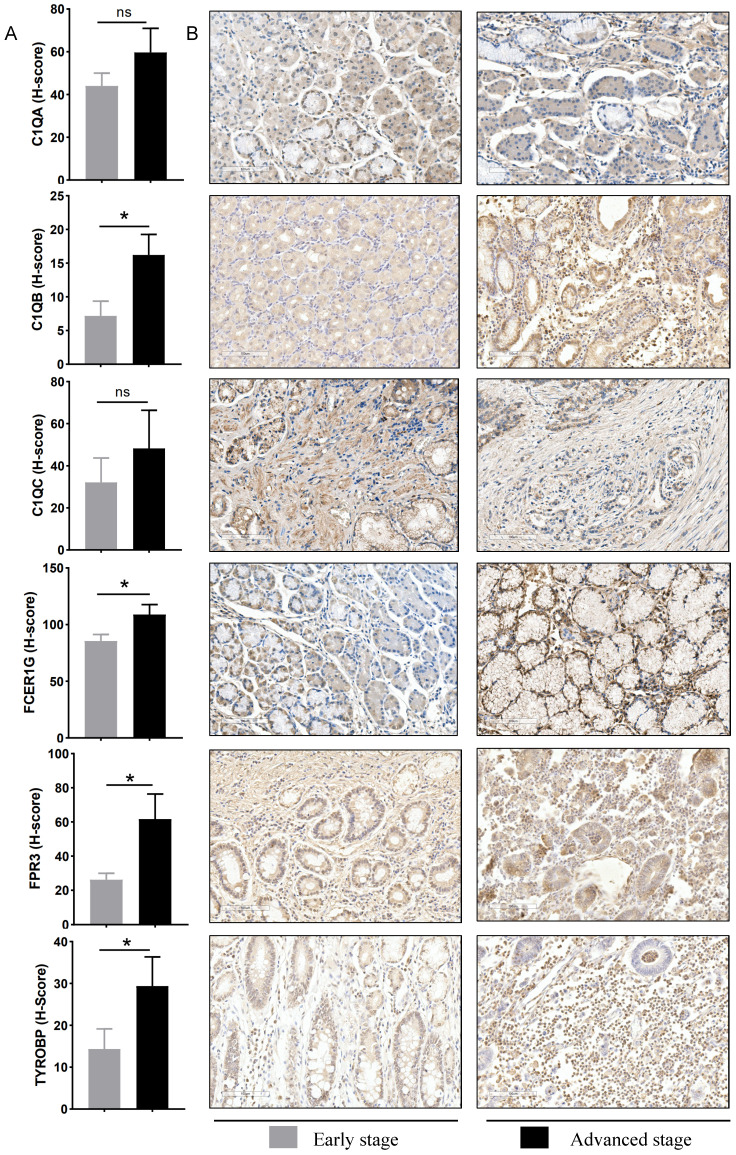
Experimental verification of six hub genes in different gastric cancer stage tissues. (A) Statistical analysis of immunohistochemistry results in early-stage tissues (n = 9) and advanced stage tissues (n = 9). Data were assessed via the method of Mann-Whitney U test. * *p*-value < 0.05, ns, no significance. (B) Representative images of immunohistochemical staining for *C1QA*,* C1QB*, *C1QC*, *FCER1G*, *FPR3*, and *TYROBP* between early stage and advanced stage gastric cancer patients. Scale bars = 100 μm.

To verify the bioinformatics results, IHC experiments were further conducted. The results revealed that the protein expression levels of *C1QB* (*P*=0.024), *FCER1G* (*P*=0.019), *FPR3* (*P*=0.024) and *TYROBP* (*P*=0.038) were significantly higher in the advanced stage group. The difference in the *C1QA* (*P*=0.354) and *C1QC* (*P*=0.402) expression between the two groups was not statistically significant (**Figure 7**). These finding indicated that the results of data mining were reliable and had potential research value.

## 4. Discussion

GC remains one of the world's most malignant cancers with heterogeneous characteristics. Although some studies have explored molecular and function related to its pathogenesis, diagnosis and prognosis, they are not sufficient to elucidate GC clearly. Therefore, we performed WGCNA analysis, GO and KEGG enrichment analysis, GSEA analysis through GC-related data in the GEO database, and through OS analysis, TCGA database analysis and IHC verification, we found that *C1QB*, *FCER1G*, *FPR3* and *TYROBP* may be the core genes for the occurrence and development of GC, and their mechanism of action may involve toll-like receptor signaling pathway, neuroactive ligand-receptor interaction, lysosome, gastric acid secretion, cholesterol metabolism, INFγ response activation and oxidative phosphorylation suppression.

Through WGCNA, we identified modules likely to be important in GC development, whose genes were strongly related to toll-like receptor signaling pathway, cholesterol metabolism, INFγ response activation and oxidative phosphorylation suppression. Consistent with the predicted functional enrichment of genes in significant modules, the toll-like receptor signaling pathway has been shown to be associated with the pathogenesis of immune diseases and cancer through stimulating several downstream signaling pathways 13, 14. Besides, toll-like receptor signaling pathway involved gene such as TLR4 expression is linked to several cancers. Huang et al. have reported that TLR4 is expressed in mouse tumor cells and activation of TLR4 in these cells induces the expression of a variety of soluble factors, making tumor cells resistant to Cytotoxic T lymphocyte attacks 15. Additionally, toll-like receptors play a major role in early innate immune defense mechanisms through activating canonical and non-canonical pathways of inflammation 16. Our study also highlighted the dysregulation of the toll-like receptors signaling pathway in the progression of GC and the differences in the composition of some innate immune system cells, such as T cells, NK cells, and macrophages, which indicated that the toll-like receptors signaling pathway and innate immune system regulation were involved in the occurrence and development of GC. In general, cholesterol and its metabolites (precursors and derivatives) play complex roles in tumors. In recent years, studies have reported the role of cholesterol metabolism in regulating tumor biological processes, especially carcinogenic signaling pathways and tumor microenvironment 17, 18. Preclinical studies over the years have shown that manipulating the synthesis and uptake of cholesterol has an inhibitory effect on tumor growth, invasion and proliferation in a variety of cancers 18-20. In addition, some new cholesterol metabolism molecules, such as SOAT1, SQLE and NPC1, have recently become promising drug targets for cancer treatment 21. Some IFNγ signaling related genes, such as IRF family genes, play an important role in the prognosis of RCC by regulating cell cycle and inducing apoptosis 22. Tumor cells are the key responders of IFN-γ in the tumor microenvironment 23. The immune activation of IFN-γ on tumor cells is mainly attributed to the induction of tumor cells to express MHC class I, secrete chemokines, promote lymphocyte migration, and inhibit angiogenesis 24-26. By directly killing gastric parietal cells, IFN-γ also appears to be a driver of disease progression to metaplasia during chronic gastritis 27.

The TME is a highly structured ecosystem that includes a rich diversity of immune cells, cancer-associated fibroblasts, endothelial cells, pericytes, and other cell types that vary from tissue to tissue, such as fat cells and neurons 28-30. As a key element in the evolutionary and ecological processes of tumorigenesis and treatment, the TME has received increasing attention in research and drug development. Previous studies have shown that high levels of immune cell infiltration are associated with a better prognosis for prostate cancer, cutaneous melanoma, and breast cancer. And increasing evidence indicated the implication the profound effects of TME in tumorigenesis, progression, and therapeutic resistance of cancers 31-33. Researches have revealed that that inhibitory Tregs, tumor-associated stromal cells (TASCs), tumor-associated macrophages (TAMs), Tc17, and CD8+ depleted T cells are enriched in the tumor, while mast cells, endocrine, and follicular regulatory T cells are enriched in paracancer tissue through a comprehensive GC single cell transcriptome map. Notably, a higher proportion of TASCs was associated with worse prognosis 34. Similarly, our analysis of TME landscape highlighted the enrichment of macrophages M1, macrophages M2 and follicular helper T cells in advanced stage tumor tissues while the levels of B cell naive, T cells CD4 memory resting, and NK cells resting were significantly decreased compared with that in early stage. Studies have proved that macrophages are players in the innate immune response and a major component of the leukocyte infiltrate present in solid tumors, which have two activated polarized states, namely classical (M1 polarization with tumor inhibitory function) and alternative (M2 polarization with tumor promoting and immunosuppression function) 35, 36. Additionally, inducing M2 polarization and inhibiting M1 polarization in the TME of GC is one of the important factors in the formation of immune tolerance 36, 37. We found higher distribution of both macrophages M1 and macrophages M2 in advanced stage patients. This may be due to the increase of M1 macrophages in tumor tissues of advanced patients to inhibit tumor growth, while the balance of TME in advanced tissues was destroyed, and M2 macrophages also increased and promoted tumor growth. NK cells can directly kill target cells and recognize tumor cells that CD8 + T cannot recognize. In GC patients, the infiltration level of NK cells in tumors and the infiltration level of NK cells in peripheral blood were positively correlated with the prognosis 38, 39. Although the cytotoxicity of resting NK cells to GC is very low, NK cells induced by K562-mb15-41BBL cell line in vitro have strong cytotoxicity to GC and showed strong anti-tumor activity in animal experiments 40. Our findings in this study were consisted with these results.

In the present study, WGCNA networks revealed four common hub genes between early stage and advanced stage of GC patients, including *C1QB*, *FCER1G*, *FPR3* and *TYROBP*. And these hub genes were strongly correlated with overall survival of GC patients. Previous studies have shown that C1q is the first recognition subcomponent of the complement classical pathway, which is comprised of three chains: C1qA, C1qB, and C1qC, and plays complex effects in the occurrence of various tumors, such as prostate cancer 41, ovarian cancer 42 and gliomas 43. According to Yamada's report 44, high expressed *C1QB* was significantly related to poor prognosis in renal cell carcinoma. A similar scenario was also observed in the KM analysis of GC patients that *C1QB* was negatively associated with prognosis of patients. *FCER1G* is located on chromosome 1q23.3 and encodes the γ subunit of the crystalline (Fc) region (Fc R) of an immunoglobulin fragment, which participates in various immune responses such as phagocytosis and cytokine release 45, 46. *FCER1G* appears to be altered in the progression of several cancers, such as esophageal squamous cell carcinoma, multiple myeloma, and clear cell renal cell carcinoma 46-48. In addition, *FCER1G* has been linked to macrophage and T cell function in renal cancer 48. These findings were consistent with our results that high expression of *FCER1G* was positively correlated with infiltration of M2 macrophages and negatively correlated with CD4 T cells, NK cells. Formyl-peptide receptors (FPRs) belong to the classical GPCR subfamily and three FPRs has been identified in humans: *FPR1*, *FPR2* and *FPR3*. It has been reported that *FPR1* and *FPR2* are abnormally expressed in various tumors 49. The expression of *FPR1* in GC tissues is higher than that in normal tissues, which is closely related to the survival time of patients 50, and *FPR2* is also highly expressed in endometrial and colon cancer 51. However, little research has been done on *FPR3*. *FPR3* is expressed in eosinophils, monocytes, macrophages, and dendritic cells, but its function is unclear 52. Several ligands for *FPR3* have been identified, including F2L, the acetylated n-terminal fragment of human heme-binding protein 53, and the neuroprotective peptide humanin 54. Interestingly, *FPR3* does not interact with formylated chemoattractants or ligands of *FPR1* or *FPR2*. Therefore, *FPR3* may have a unique functional role 55. A recent study identified *FPR3* as a key immune-related biomarker for predicting poor prognosis of breast cancer, possibly playing an important role in the progression of breast cancer by modulating the immune microenvironment 56. Our study demonstrated the important role of *FPR3* in GC progression. Moreover, our results revealed a positive correlation between *TYROBP* and tumor stage in GC. *TYROBP*, also known as *DAP12* is notably positive with tumor progression in multiple cancers. Previous study has uncovered an association between high *TYROBP* expression with high-risk metastases as well as poor survival of breast cancer patients 57. In our present study, *TYROBP* overexpression was associated with tumor stage and poor survival of GC patients. Additionally, results from interrelation analysis showed that *TYROBP* was positively associated with macrophage M2 and CD8 T cells, which negatively correlated with NK cells resting. This is consistent with some previous studies 58, 59. These results indicated that *TYROBP* might be playing an immunosuppressive role to promote tumor immune escape in advanced GC.

The current study also has several limitations. Firstly, this study was based on bioinformatics analysis, and a relatively small cohort was recruited for hub genes verification, so further validation using large clinical cohorts should be performed to verify these results. Secondly, the results lacked vitro and vivo exploration to confirm the reliability of the mechanistic analysis. Therefore, a number of experiments should be conducted to demonstrate the mechanistic connections between these genes and GC progression in the future.

## 5. Conclusion

In conclusion, the current study identified 4 hub genes associated with GC progression and patients' prognosis through a series of bioinformatics analyses and validation. In addition, the four hub genes were significantly correlated with the infiltration of immune cells, which may help guide further studies to gain a comprehensive understanding of the network of hub genes involved in tumor progression and provide valuable clues for the treatment of GC.

## Supplementary Material

Supplementary figures and tables.
