# Supplementary material for: Identification of crucial genes through WGCNA in the progression of gastric cancer
Source: J Cancer. 2024 Apr 23;15(11):3284–96. doi: 10.7150/jca.95757 (PMC11134444; doi:10.7150/jca.95757)
Supplement: Supplementary file 1 — Supplementary figures and tables. [file jcav15p3284s1.pdf]

Supplementary file 1:

Patient ID	Age	Sex(1=male; 0=female)	Stage
1	65	1	1
2	76	1	2
3	66	0	2
4	59	0	2
5	71	0	1
6	58	1	2
7	76	1	2
8	55	0	1
9	80	1	2
10	77	1	2
11	69	0	3
12	73	0	3
13	79	0	3
14	64	1	3
15	65	0	3
16	82	1	3
17	54	1	4
18	78	1	3
19	58	1	4
20	65	0	3

Supplementary file 2:

Term ID	Category	Term description	false discovery rate
GO:0016021	Cellular Component	Integral component of membrane	8.43E-19
GO:0031224	Cellular Component	Intrinsic component of membrane	1.01E-18
GO:0031226	Cellular Component	Intrinsic component of plasma membrane	6.04E-15
GO:0005887	Cellular Component	Integral component of plasma membrane	8.84E-14
GO:0005886	Cellular Component	Plasma membrane	1.64E-12
GO:0071944	Cellular Component	Cell periphery	1.64E-12
GO:0031410	Cellular Component	Cytoplasmic vesicle	1.94E-12

GO:0030667	Cellular Component	Secretory granule membrane	2.59E-11
GO:0098805	Cellular Component	Whole membrane	1.22E-10
GO:0030141	Cellular Component	Secretory granule	3.17E-10
GO:0016020	Cellular Component	Membrane	4.36E-10
GO:0030659	Cellular Component	Cytoplasmic vesicle membrane	8.57E-10
GO:0099503	Cellular Component	Secretory vesicle	3.99E-09
GO:0031982	Cellular Component	Vesicle	5.81E-09
GO:0070821	Cellular Component	Tertiary granule membrane	8.11E-09
GO:0009986	Cellular Component	Cell surface	1.54E-08
GO:0098588	Cellular Component	Bounding membrane of organelle	5.74E-08
GO:0070820	Cellular Component	Tertiary granule	1.83E-07
GO:0012505	Cellular Component	Endomembrane system	1.55E-06
GO:0009897	Cellular Component	External side of plasma membrane	1.74E-06
GO:0098552	Cellular Component	Side of membrane	3.56E-05
GO:0005773	Cellular Component	Vacuole	4.31E-05
GO:0005764	Cellular Component	Lysosome	9.17E-05
GO:0036019	Cellular Component	Endolysosome	0.00015
GO:0005768	Cellular Component	Endosome	0.00023
GO:0035579	Cellular Component	Specific granule membrane	0.00097
GO:0005775	Cellular Component	Vacuolar lumen	0.0012
GO:0101003	Cellular Component	ficolin-1-rich granule membrane	0.0012
GO:0043202	Cellular Component	Lysosomal lumen	0.0013
GO:0036021	Cellular Component	Endolysosome lumen	0.0019

GO:0046696	Cellular Component	Lipopolysaccharide receptor complex	0.0019
GO:0030139	Cellular Component	Endocytic vesicle	0.0026
GO:0031090	Cellular Component	Organelle membrane	0.0027
GO:0110165	Cellular Component	Cellular anatomical entity	0.0036
GO:0045335	Cellular Component	Phagocytic vesicle	0.0093
GO:0005576	Cellular Component	Extracellular region	0.0096
GO:0010008	Cellular Component	Endosome membrane	0.0128
GO:0034362	Cellular Component	Low-density lipoprotein particle	0.0148
GO:0030666	Cellular Component	Endocytic vesicle membrane	0.0186
GO:0034358	Cellular Component	Plasma lipoprotein particle	0.0186
GO:0045121	Cellular Component	Membrane raft	0.0186
GO:0005602	Cellular Component	Complement component c1 complex	0.0187
GO:0034688	Cellular Component	Integrin alpham-beta2 complex	0.0187
GO:0034689	Cellular Component	Integrin alphax-beta2 complex	0.0187
GO:0035354	Cellular Component	Toll-like receptor 1-Toll-like receptor 2 protein complex	0.0187
GO:0005615	Cellular Component	Extracellular space	0.0445
GO:0043235	Cellular Component	Receptor complex	0.048
GO:0038023	Molecular Function	Signaling receptor activity	7.84E-12
GO:0038187	Molecular Function	Pattern recognition receptor activity	1.30E-10
GO:0001540	Molecular Function	Amyloid-beta binding	1.86E-06
GO:0033218	Molecular Function	Amide binding	1.11E-05
GO:0004888	Molecular Function	Transmembrane signaling receptor activity	1.20E-05
GO:0001530	Molecular Function	Lipopolysaccharide binding	2.17E-05

GO:0019864	Molecular Function	IgG binding	5.17E-05
GO:0019865	Molecular Function	Immunoglobulin binding	5.17E-05
GO:0042277	Molecular Function	Peptide binding	9.60E-05
GO:0140375	Molecular Function	Immune receptor activity	9.60E-05
GO:0001875	Molecular Function	Lipopolysaccharide immune receptor activity	0.0002
GO:0004875	Molecular Function	Complement receptor activity	0.0019
GO:0008289	Molecular Function	Lipid binding	0.0048
GO:0001851	Molecular Function	Complement component c3b binding	0.009
GO:0001848	Molecular Function	Complement binding	0.0126
GO:0019763	Molecular Function	Immunoglobulin receptor activity	0.0126
GO:0033691	Molecular Function	Sialic acid binding	0.0139
GO:0071723	Molecular Function	Lipopeptide binding	0.0353
GO:0005215	Molecular Function	Transporter activity	0.0483
GO:0035325	Molecular Function	Toll-like receptor binding	0.0493
GO:0042287	Molecular Function	MHC protein binding	0.0493
GO:0006955	Biological Process	Immune response	5.61E-23
GO:0002376	Biological Process	Immune system process	2.28E-21
GO:0006952	Biological Process	Defense response	5.10E-20
GO:0050776	Biological Process	Regulation of immune response	9.12E-20
GO:0002682	Biological Process	Regulation of immune system process	1.19E-19
GO:0006954	Biological Process	Inflammatory response	1.19E-19
GO:0002684	Biological Process	Positive regulation of immune system process	3.68E-18
GO:0045321	Biological Process	Leukocyte activation	1.10E-17

GO:0002274	Biological Process	Myeloid leukocyte activation	3.97E-17
GO:0051707	Biological Process	Response to other organism	1.20E-14
GO:1903555	Biological Process	Regulation of tumor necrosis factor superfamily cytokine production	1.20E-14
GO:0001817	Biological Process	Regulation of cytokine production	3.00E-14
GO:0002252	Biological Process	Immune effector process	5.30E-14
GO:0002366	Biological Process	Leukocyte activation involved in immune response	8.66E-14
GO:0032680	Biological Process	Regulation of tumor necrosis factor production	9.13E-14
GO:0098542	Biological Process	Defense response to other organism	1.66E-13
GO:0050865	Biological Process	Regulation of cell activation	3.67E-13
GO:0002221	Biological Process	Pattern recognition receptor signaling pathway	9.42E-13
GO:0001819	Biological Process	Positive regulation of cytokine production	9.59E-13
GO:0045087	Biological Process	Innate immune response	1.42E-12
GO:0002250	Biological Process	Adaptive immune response	1.94E-12
GO:0002275	Biological Process	Myeloid cell activation involved in immune response	1.95E-12
GO:0042116	Biological Process	Macrophage activation	2.43E-12
GO:0009605	Biological Process	Response to external stimulus	3.62E-12
GO:0002443	Biological Process	Leukocyte mediated immunity	5.03E-12
GO:0050778	Biological Process	Positive regulation of immune response	8.77E-12
GO:0002224	Biological Process	Toll-like receptor signaling pathway	9.34E-12
GO:0002694	Biological Process	Regulation of leukocyte activation	1.73E-11
GO:0002283	Biological Process	Neutrophil activation involved in immune response	1.96E-11
GO:0140352	Biological Process	Export from cell	2.97E-11
GO:0043299	Biological Process	Leukocyte degranulation	3.88E-11

GO:1903557	Biological Process	Positive regulation of tumor necrosis factor superfamily cytokine production	3.88E-11
GO:0032675	Biological Process	Regulation of interleukin-6 production	5.48E-11
GO:0032755	Biological Process	Positive regulation of interleukin-6 production	6.13E-11
GO:0016192	Biological Process	Vesicle-mediated transport	7.03E-11
GO:0043312	Biological Process	Neutrophil degranulation	9.75E-11
GO:0044419	Biological Process	Interspecies interaction between organisms	9.91E-11
GO:0046903	Biological Process	Secretion	1.50E-10
GO:0045055	Biological Process	Regulated exocytosis	1.62E-10
GO:0071216	Biological Process	Cellular response to biotic stimulus	1.85E-10
GO:0002237	Biological Process	Response to molecule of bacterial origin	1.96E-10
GO:0032103	Biological Process	Positive regulation of response to external stimulus	2.63E-10
GO:0001774	Biological Process	Microglial cell activation	2.79E-10
GO:0071219	Biological Process	Cellular response to molecule of bacterial origin	3.13E-10
GO:0032101	Biological Process	Regulation of response to external stimulus	3.51E-10
GO:0032760	Biological Process	Positive regulation of tumor necrosis factor production	3.76E-10
GO:0006887	Biological Process	Exocytosis	4.53E-10
GO:0032879	Biological Process	Regulation of localization	5.70E-10
GO:0032940	Biological Process	Secretion by cell	6.71E-10
GO:0006950	Biological Process	Response to stress	8.94E-10
GO:0032652	Biological Process	Regulation of interleukin-1 production	1.33E-09
GO:0002697	Biological Process	Regulation of immune effector process	1.48E-09
GO:0050672	Biological Process	Negative regulation of lymphocyte proliferation	2.09E-09

GO:0006810	Biological Process	Transport	2.68E-09
GO:0032651	Biological Process	Regulation of interleukin-1 beta production	3.79E-09
GO:0032496	Biological Process	Response to lipopolysaccharide	4.15E-09
GO:0050866	Biological Process	Negative regulation of cell activation	4.46E-09
GO:0006909	Biological Process	Phagocytosis	7.47E-09
GO:0002253	Biological Process	Activation of immune response	1.55E-08
GO:0009617	Biological Process	Response to bacterium	1.55E-08
GO:0050896	Biological Process	Response to stimulus	1.69E-08
GO:0098581	Biological Process	Detection of external biotic stimulus	1.89E-08
GO:0032653	Biological Process	Regulation of interleukin-10 production	2.58E-08
GO:0051179	Biological Process	Localization	4.90E-08
GO:0031347	Biological Process	Regulation of defense response	4.93E-08
GO:0032732	Biological Process	Positive regulation of interleukin-1 production	5.97E-08
GO:0048584	Biological Process	Positive regulation of response to stimulus	7.78E-08
GO:0051239	Biological Process	Regulation of multicellular organismal process	8.34E-08
GO:0002695	Biological Process	Negative regulation of leukocyte activation	9.64E-08
GO:0045088	Biological Process	Regulation of innate immune response	1.20E-07
GO:0071222	Biological Process	Cellular response to lipopolysaccharide	1.23E-07
GO:0050867	Biological Process	Positive regulation of cell activation	1.24E-07
GO:0002764	Biological Process	Immune response-regulating signaling pathway	1.40E-07
GO:0002683	Biological Process	Negative regulation of immune system process	1.75E-07
GO:0051049	Biological Process	Regulation of transport	2.49E-07
GO:0031349	Biological Process	Positive regulation of defense response	2.87E-07

GO:0048583	Biological Process	Regulation of response to stimulus	2.87E-07
GO:0032731	Biological Process	Positive regulation of interleukin-1 beta production	3.25E-07
GO:0002696	Biological Process	Positive regulation of leukocyte activation	4.40E-07
GO:0032490	Biological Process	Detection of molecule of bacterial origin	4.54E-07
GO:0002703	Biological Process	Regulation of leukocyte mediated immunity	4.79E-07
GO:0002886	Biological Process	Regulation of myeloid leukocyte mediated immunity	4.79E-07
GO:0002768	Biological Process	Immune response-regulating cell surface receptor signaling pathway	7.62E-07
GO:0050670	Biological Process	Regulation of lymphocyte proliferation	7.76E-07
GO:0002831	Biological Process	Regulation of response to biotic stimulus	1.17E-06
GO:0070887	Biological Process	Cellular response to chemical stimulus	1.17E-06
GO:0007166	Biological Process	Cell surface receptor signaling pathway	1.58E-06
GO:0043300	Biological Process	Regulation of leukocyte degranulation	1.80E-06
GO:0001818	Biological Process	Negative regulation of cytokine production	2.01E-06
GO:0051240	Biological Process	Positive regulation of multicellular organismal process	2.15E-06
GO:0032677	Biological Process	Regulation of interleukin-8 production	3.60E-06
GO:0002755	Biological Process	MyD88-dependent toll-like receptor signaling pathway	3.86E-06
GO:0098543	Biological Process	Detection of other organism	4.04E-06
GO:0051249	Biological Process	Regulation of lymphocyte activation	4.10E-06
GO:0032757	Biological Process	Positive regulation of interleukin-8 production	5.05E-06
GO:0042221	Biological Process	Response to chemical	5.12E-06
GO:1903530	Biological Process	Regulation of secretion by cell	6.44E-06
GO:0034142	Biological Process	Toll-like receptor 4 signaling pathway	6.48E-06

GO:0051241	Biological Process	Negative regulation of multicellular organismal process	6.48E-06
GO:0002699	Biological Process	Positive regulation of immune effector process	7.00E-06
GO:0010033	Biological Process	Response to organic substance	7.99E-06
GO:0032720	Biological Process	Negative regulation of tumor necrosis factor production	8.79E-06
GO:0001816	Biological Process	Cytokine production	1.41E-05
GO:0051050	Biological Process	Positive regulation of transport	1.41E-05
GO:0006911	Biological Process	Phagocytosis, engulfment	1.72E-05
GO:0034121	Biological Process	Regulation of toll-like receptor signaling pathway	2.26E-05
GO:0050727	Biological Process	Regulation of inflammatory response	2.86E-05
GO:0043269	Biological Process	Regulation of ion transport	2.92E-05
GO:1902563	Biological Process	Regulation of neutrophil activation	3.01E-05
GO:0002429	Biological Process	Immune response-activating cell surface receptor signaling pathway	3.98E-05
GO:0097242	Biological Process	Amyloid-beta clearance	3.98E-05
GO:1903532	Biological Process	Positive regulation of secretion by cell	4.04E-05
GO:1903037	Biological Process	Regulation of leukocyte cell-cell adhesion	4.48E-05
GO:0071310	Biological Process	Cellular response to organic substance	5.04E-05
GO:0033993	Biological Process	Response to lipid	5.23E-05
GO:0006898	Biological Process	Receptor-mediated endocytosis	5.30E-05
GO:0071675	Biological Process	Regulation of mononuclear cell migration	5.55E-05
GO:1901224	Biological Process	Positive regulation of nik/nf-kappab signaling	6.83E-05
GO:0007165	Biological Process	Signal transduction	7.03E-05
GO:0032642	Biological Process	Regulation of chemokine production	7.97E-05

GO:0031663	Biological Process	Lipopolysaccharide-mediated signaling pathway	8.06E-05
GO:0050868	Biological Process	Negative regulation of t cell activation	8.29E-05
GO:0023052	Biological Process	Signaling	9.20E-05
GO:0051716	Biological Process	Cellular response to stimulus	9.90E-05
GO:0002833	Biological Process	Positive regulation of response to biotic stimulus	0.00012
GO:0032722	Biological Process	Positive regulation of chemokine production	0.00012
GO:0050764	Biological Process	Regulation of phagocytosis	0.00014
GO:0032733	Biological Process	Positive regulation of interleukin-10 production	0.00015
GO:0034122	Biological Process	Negative regulation of toll-like receptor signaling pathway	0.00015
GO:0045089	Biological Process	Positive regulation of innate immune response	0.00015
GO:0002685	Biological Process	Regulation of leukocyte migration	0.00016
GO:0007154	Biological Process	Cell communication	0.00016
GO:0042130	Biological Process	Negative regulation of t cell proliferation	0.00019
GO:0045670	Biological Process	Regulation of osteoclast differentiation	0.0002
GO:1901216	Biological Process	Positive regulation of neuron death	0.00021
GO:0022407	Biological Process	Regulation of cell-cell adhesion	0.00022
GO:1901700	Biological Process	Response to oxygen-containing compound	0.00023
GO:0032928	Biological Process	Regulation of superoxide anion generation	0.00025
GO:0060627	Biological Process	Regulation of vesicle-mediated transport	0.00026
GO:0006897	Biological Process	Endocytosis	0.00028
GO:0002218	Biological Process	Activation of innate immune response	0.00032
GO:0022408	Biological Process	Negative regulation of cell-cell adhesion	0.00032
GO:0042742	Biological Process	Defense response to bacterium	0.00033

GO:0032649	Biological Process	Regulation of interferon-gamma production	0.00034
GO:0002687	Biological Process	Positive regulation of leukocyte migration	0.00035
GO:0007162	Biological Process	Negative regulation of cell adhesion	0.00035
GO:0050729	Biological Process	Positive regulation of inflammatory response	0.00035
GO:0008285	Biological Process	Negative regulation of cell population proliferation	0.00036
GO:0002920	Biological Process	Regulation of humoral immune response	0.00038
GO:0097006	Biological Process	Regulation of plasma lipoprotein particle levels	0.00038
GO:1901701	Biological Process	Cellular response to oxygen-containing compound	0.00038
GO:0009987	Biological Process	Cellular process	0.00039
GO:1902105	Biological Process	Regulation of leukocyte differentiation	0.00041
GO:0048002	Biological Process	Antigen processing and presentation of peptide antigen	0.00043
GO:0071223	Biological Process	Cellular response to lipoteichoic acid	0.00045
GO:0090025	Biological Process	Regulation of monocyte chemotaxis	0.00053
GO:0043085	Biological Process	Positive regulation of catalytic activity	0.00062
GO:0065008	Biological Process	Regulation of biological quality	0.00066
GO:0006968	Biological Process	Cellular defense response	0.0007
GO:0034097	Biological Process	Response to cytokine	0.00071
GO:0044093	Biological Process	Positive regulation of molecular function	0.00071
GO:0002761	Biological Process	Regulation of myeloid leukocyte differentiation	0.00076
GO:0043030	Biological Process	Regulation of macrophage activation	0.00076
GO:0030449	Biological Process	Regulation of complement activation	0.00083
GO:0070374	Biological Process	Positive regulation of erk1 and erk2 cascade	0.00084
GO:0071727	Biological Process	Cellular response to triacyl bacterial lipopeptide	0.00084

GO:2000363	Biological Process	Positive regulation of prostaglandin-e synthase activity	0.00084
GO:0002688	Biological Process	Regulation of leukocyte chemotaxis	0.00086
GO:0002756	Biological Process	MyD88-independent toll-like receptor signaling pathway	0.001
GO:1901214	Biological Process	Regulation of neuron death	0.001
GO:0031341	Biological Process	Regulation of cell killing	0.0011
GO:0032655	Biological Process	Regulation of interleukin-12 production	0.0012
GO:0002478	Biological Process	Antigen processing and presentation of exogenous peptide antigen	0.0013
GO:0002888	Biological Process	Positive regulation of myeloid leukocyte mediated immunity	0.0013
GO:0007229	Biological Process	Integrin-mediated signaling pathway	0.0013
GO:0007155	Biological Process	Cell adhesion	0.0014
GO:0016045	Biological Process	Detection of bacterium	0.0014
GO:0032497	Biological Process	Detection of lipopolysaccharide	0.0014
GO:0038123	Biological Process	Toll-like receptor tlr1:tlr2 signaling pathway	0.0014
GO:0043315	Biological Process	Positive regulation of neutrophil degranulation	0.0014
GO:0050863	Biological Process	Regulation of t cell activation	0.0014
GO:0002690	Biological Process	Positive regulation of leukocyte chemotaxis	0.0016
GO:0030889	Biological Process	Negative regulation of b cell proliferation	0.0016
GO:0071345	Biological Process	Cellular response to cytokine stimulus	0.0016
GO:1903039	Biological Process	Positive regulation of leukocyte cell-cell adhesion	0.0016
GO:0030334	Biological Process	Regulation of cell migration	0.0017
GO:0051251	Biological Process	Positive regulation of lymphocyte activation	0.0019
GO:0071396	Biological Process	Cellular response to lipid	0.002

GO:0034381	Biological Process	Plasma lipoprotein particle clearance	0.0021
GO:0080134	Biological Process	Regulation of response to stress	0.0021
GO:0015850	Biological Process	Organic hydroxy compound transport	0.0022
GO:0032693	Biological Process	Negative regulation of interleukin-10 production	0.0022
GO:0050765	Biological Process	Negative regulation of phagocytosis	0.0022
GO:0032729	Biological Process	Positive regulation of interferon-gamma production	0.0023
GO:0050921	Biological Process	Positive regulation of chemotaxis	0.0024
GO:0002460	Biological Process	Adaptive immune response based on somatic recombination of immune receptors built from immunoglobulin superfamily domains	0.0025
GO:0002577	Biological Process	Regulation of antigen processing and presentation	0.0026
GO:0032930	Biological Process	Positive regulation of superoxide anion generation	0.0026
GO:0033003	Biological Process	Regulation of mast cell activation	0.0027
GO:1990573	Biological Process	Potassium ion import across plasma membrane	0.0027
GO:0001910	Biological Process	Regulation of leukocyte mediated cytotoxicity	0.0029
GO:0007249	Biological Process	I-kappaB kinase/NF-kappaB signaling	0.0029
GO:0090026	Biological Process	Positive regulation of monocyte chemotaxis	0.003
GO:0050790	Biological Process	Regulation of catalytic activity	0.0034
GO:0051924	Biological Process	Regulation of calcium ion transport	0.0036
GO:0034128	Biological Process	Negative regulation of myd88-independent toll-like receptor signaling pathway	0.0039
GO:0033344	Biological Process	Cholesterol efflux	0.004
GO:0042592	Biological Process	Homeostatic process	0.004
GO:0048518	Biological Process	Positive regulation of biological process	0.004
GO:0050900	Biological Process	Leukocyte migration	0.004

GO:0071346	Biological Process	Cellular response to interferon-gamma	0.004
GO:0030155	Biological Process	Regulation of cell adhesion	0.0043
GO:0098657	Biological Process	Import into cell	0.0045
GO:0042129	Biological Process	Regulation of t cell proliferation	0.0047
GO:0016064	Biological Process	Immunoglobulin mediated immune response	0.0049
GO:0002765	Biological Process	Immune response-inhibiting signal transduction	0.0051
GO:0043032	Biological Process	Positive regulation of macrophage activation	0.0051
GO:0050714	Biological Process	Positive regulation of protein secretion	0.0051
GO:0098883	Biological Process	Synapse pruning	0.0051
GO:0002698	Biological Process	Negative regulation of immune effector process	0.0055
GO:0002704	Biological Process	Negative regulation of leukocyte mediated immunity	0.0055
GO:0002762	Biological Process	Negative regulation of myeloid leukocyte differentiation	0.0055
GO:0010959	Biological Process	Regulation of metal ion transport	0.0055
GO:0032648	Biological Process	Regulation of interferon-beta production	0.0055
GO:0043302	Biological Process	Positive regulation of leukocyte degranulation	0.0055
GO:0065007	Biological Process	Biological regulation	0.0055
GO:0050920	Biological Process	Regulation of chemotaxis	0.006
GO:0050864	Biological Process	Regulation of b cell activation	0.0062
GO:0051649	Biological Process	Establishment of localization in cell	0.0062
GO:0030301	Biological Process	Cholesterol transport	0.0063
GO:0042127	Biological Process	Regulation of cell population proliferation	0.0063
GO:0019725	Biological Process	Cellular homeostasis	0.0065
GO:0032479	Biological Process	Regulation of type i interferon production	0.0065

GO:0032691	Biological Process	Negative regulation of interleukin-1 beta production	0.0068
GO:0043270	Biological Process	Positive regulation of ion transport	0.0072
GO:0035590	Biological Process	Purinergic nucleotide receptor signaling pathway	0.0077
GO:0045671	Biological Process	Negative regulation of osteoclast differentiation	0.0077
GO:0002887	Biological Process	Negative regulation of myeloid leukocyte mediated immunity	0.0078
GO:0035666	Biological Process	TRIF-dependent toll-like receptor signaling pathway	0.0085
GO:0002705	Biological Process	Positive regulation of leukocyte mediated immunity	0.0086
GO:0048871	Biological Process	Multicellular organismal homeostasis	0.0086
GO:0051222	Biological Process	Positive regulation of protein transport	0.0086
GO:1903426	Biological Process	Regulation of reactive oxygen species biosynthetic process	0.0087
GO:0043304	Biological Process	Regulation of mast cell degranulation	0.0094
GO:2000377	Biological Process	Regulation of reactive oxygen species metabolic process	0.0096
GO:0002449	Biological Process	Lymphocyte mediated immunity	0.0099
GO:0007200	Biological Process	Phospholipase c-activating g protein-coupled receptor signaling pathway	0.0099
GO:0045428	Biological Process	Regulation of nitric oxide biosynthetic process	0.0099
GO:0030595	Biological Process	Leukocyte chemotaxis	0.0102
GO:0032647	Biological Process	Regulation of interferon-alpha production	0.0102
GO:0032102	Biological Process	Negative regulation of response to external stimulus	0.0115
GO:0032494	Biological Process	Response to peptidoglycan	0.0115
GO:0033004	Biological Process	Negative regulation of mast cell activation	0.0115
GO:0043301	Biological Process	Negative regulation of leukocyte degranulation	0.0115
GO:2001198	Biological Process	Regulation of dendritic cell differentiation	0.0115

GO:0030888	Biological Process	Regulation of b cell proliferation	0.0117
GO:0010543	Biological Process	Regulation of platelet activation	0.0122
GO:0032728	Biological Process	Positive regulation of interferon-beta production	0.0122
GO:2000379	Biological Process	Positive regulation of reactive oxygen species metabolic process	0.0123
GO:1902106	Biological Process	Negative regulation of leukocyte differentiation	0.0128
GO:0050766	Biological Process	Positive regulation of phagocytosis	0.0132
GO:0032689	Biological Process	Negative regulation of interferon-gamma production	0.0133
GO:0007252	Biological Process	I-kappaB phosphorylation	0.0135
GO:0046348	Biological Process	Amino sugar catabolic process	0.0135
GO:0051051	Biological Process	Negative regulation of transport	0.0141
GO:0050777	Biological Process	Negative regulation of immune response	0.0155
GO:0014002	Biological Process	Astrocyte development	0.0159
GO:0030335	Biological Process	Positive regulation of cell migration	0.017
GO:0050870	Biological Process	Positive regulation of t cell activation	0.0173
GO:0046649	Biological Process	Lymphocyte activation	0.018
GO:0072593	Biological Process	Reactive oxygen species metabolic process	0.0181
GO:0002281	Biological Process	Macrophage activation involved in immune response	0.0188
GO:0031348	Biological Process	Negative regulation of defense response	0.0188
GO:0050663	Biological Process	Cytokine secretion	0.0188
GO:1904646	Biological Process	Cellular response to amyloid-beta	0.0188
GO:0016046	Biological Process	Detection of fungus	0.019
GO:0032499	Biological Process	Detection of peptidoglycan	0.019
GO:0034125	Biological Process	Negative regulation of myd88-dependent toll-like	0.019

		receptor signaling pathway	
GO:0042495	Biological Process	Detection of triacyl bacterial lipopeptide	0.019
GO:0110090	Biological Process	Positive regulation of hippocampal neuron apoptotic process	0.019
GO:1904466	Biological Process	Positive regulation of matrix metallopeptidase secretion	0.019
GO:0055082	Biological Process	Cellular chemical homeostasis	0.0191
GO:0019221	Biological Process	Cytokine-mediated signaling pathway	0.0194
GO:0050793	Biological Process	Regulation of developmental process	0.0196
GO:0002758	Biological Process	Innate immune response-activating signal transduction	0.0198
GO:0050708	Biological Process	Regulation of protein secretion	0.0199
GO:0045937	Biological Process	Positive regulation of phosphate metabolic process	0.0201
GO:0002532	Biological Process	Production of molecular mediator involved in inflammatory response	0.0211
GO:0051770	Biological Process	Positive regulation of nitric-oxide synthase biosynthetic process	0.0211
GO:0006811	Biological Process	Ion transport	0.0216
GO:0038094	Biological Process	Fc-gamma receptor signaling pathway	0.0222
GO:0043410	Biological Process	Positive regulation of mapk cascade	0.0228
GO:0030316	Biological Process	Osteoclast differentiation	0.0229
GO:0098739	Biological Process	Import across plasma membrane	0.0229
GO:1905521	Biological Process	Regulation of macrophage migration	0.0229
GO:0006935	Biological Process	Chemotaxis	0.0233
GO:0034162	Biological Process	Toll-like receptor 9 signaling pathway	0.024
GO:0034765	Biological Process	Regulation of ion transmembrane transport	0.024

GO:0045766	Biological Process	Positive regulation of angiogenesis	0.024
GO:1903034	Biological Process	Regulation of response to wounding	0.024
GO:2000026	Biological Process	Regulation of multicellular organismal development	0.024
GO:0032880	Biological Process	Regulation of protein localization	0.0241
GO:0019220	Biological Process	Regulation of phosphate metabolic process	0.0246
GO:0032270	Biological Process	Positive regulation of cellular protein metabolic process	0.0254
GO:0045429	Biological Process	Positive regulation of nitric oxide biosynthetic process	0.0258
GO:0030193	Biological Process	Regulation of blood coagulation	0.0262
GO:0032481	Biological Process	Positive regulation of type i interferon production	0.0262
GO:0042590	Biological Process	Antigen processing and presentation of exogenous peptide antigen via mhc class i	0.0262
GO:0055065	Biological Process	Metal ion homeostasis	0.0264
GO:0032695	Biological Process	Negative regulation of interleukin-12 production	0.0266
GO:0045730	Biological Process	Respiratory burst	0.0266
GO:0050789	Biological Process	Regulation of biological process	0.0271
GO:0071621	Biological Process	Granulocyte chemotaxis	0.0272
GO:0001810	Biological Process	Regulation of type i hypersensitivity	0.0278
GO:0002774	Biological Process	Fc receptor mediated inhibitory signaling pathway	0.0278
GO:0034762	Biological Process	Regulation of transmembrane transport	0.0278
GO:0045963	Biological Process	Negative regulation of dopamine metabolic process	0.0278
GO:0055080	Biological Process	Cation homeostasis	0.0278
GO:1903974	Biological Process	Positive regulation of cellular response to macrophage colony-stimulating factor stimulus	0.0278
GO:1904151	Biological Process	Positive regulation of microglial cell mediated	0.0278

		cytotoxicity	
GO:2001189	Biological Process	Negative regulation of t cell activation via t cell receptor contact with antigen bound to mhc molecule on antigen presenting cell	0.0278
GO:0038093	Biological Process	Fc receptor signaling pathway	0.0284
GO:0006875	Biological Process	Cellular metal ion homeostasis	0.0292
GO:0050801	Biological Process	Ion homeostasis	0.0297
GO:0010942	Biological Process	Positive regulation of cell death	0.0301
GO:0070201	Biological Process	Regulation of establishment of protein localization	0.0301
GO:0071827	Biological Process	Plasma lipoprotein particle organization	0.0301
GO:1900271	Biological Process	Regulation of long-term synaptic potentiation	0.0301
GO:0030003	Biological Process	Cellular cation homeostasis	0.0304
GO:0098771	Biological Process	Inorganic ion homeostasis	0.0313
GO:0043408	Biological Process	Regulation of mapk cascade	0.0321
GO:0001911	Biological Process	Negative regulation of leukocyte mediated cytotoxicity	0.0322
GO:0009893	Biological Process	Positive regulation of metabolic process	0.0322
GO:0048143	Biological Process	Astrocyte activation	0.0322
GO:0061081	Biological Process	Positive regulation of myeloid leukocyte cytokine production involved in immune response	0.0322
GO:0045765	Biological Process	Regulation of angiogenesis	0.0348
GO:0050671	Biological Process	Positive regulation of lymphocyte proliferation	0.0355
GO:1900017	Biological Process	Positive regulation of cytokine production involved in inflammatory response	0.0357
GO:0051641	Biological Process	Cellular localization	0.0358
GO:0001796	Biological Process	Regulation of type iia hypersensitivity	0.0374

GO:0002282	Biological Process	Microglial cell activation involved in immune response	0.0374
GO:0002374	Biological Process	Cytokine secretion involved in immune response	0.0374
GO:0016125	Biological Process	Sterol metabolic process	0.0374
GO:0038124	Biological Process	Toll-like receptor tlr6:tlr2 signaling pathway	0.0374
GO:0071726	Biological Process	Cellular response to diacyl bacterial lipopeptide	0.0374
GO:0061041	Biological Process	Regulation of wound healing	0.0423
GO:0032753	Biological Process	Positive regulation of interleukin-4 production	0.0433
GO:0070266	Biological Process	Necroptotic process	0.0433
GO:0006956	Biological Process	Complement activation	0.0445
GO:0055074	Biological Process	Calcium ion homeostasis	0.0462
GO:0008202	Biological Process	Steroid metabolic process	0.0464
GO:0002832	Biological Process	Negative regulation of response to biotic stimulus	0.0477
GO:0019886	Biological Process	Antigen processing and presentation of exogenous peptide antigen via mhc class ii	0.0477
GO:0001932	Biological Process	Regulation of protein phosphorylation	0.049
GO:0032663	Biological Process	Regulation of interleukin-2 production	0.049
GO:0034154	Biological Process	Toll-like receptor 7 signaling pathway	0.049
GO:0034241	Biological Process	Positive regulation of macrophage fusion	0.049
GO:0042102	Biological Process	Positive regulation of t cell proliferation	0.049
GO:1901979	Biological Process	Regulation of inward rectifier potassium channel activity	0.049
GO:1902564	Biological Process	Negative regulation of neutrophil activation	0.049

4

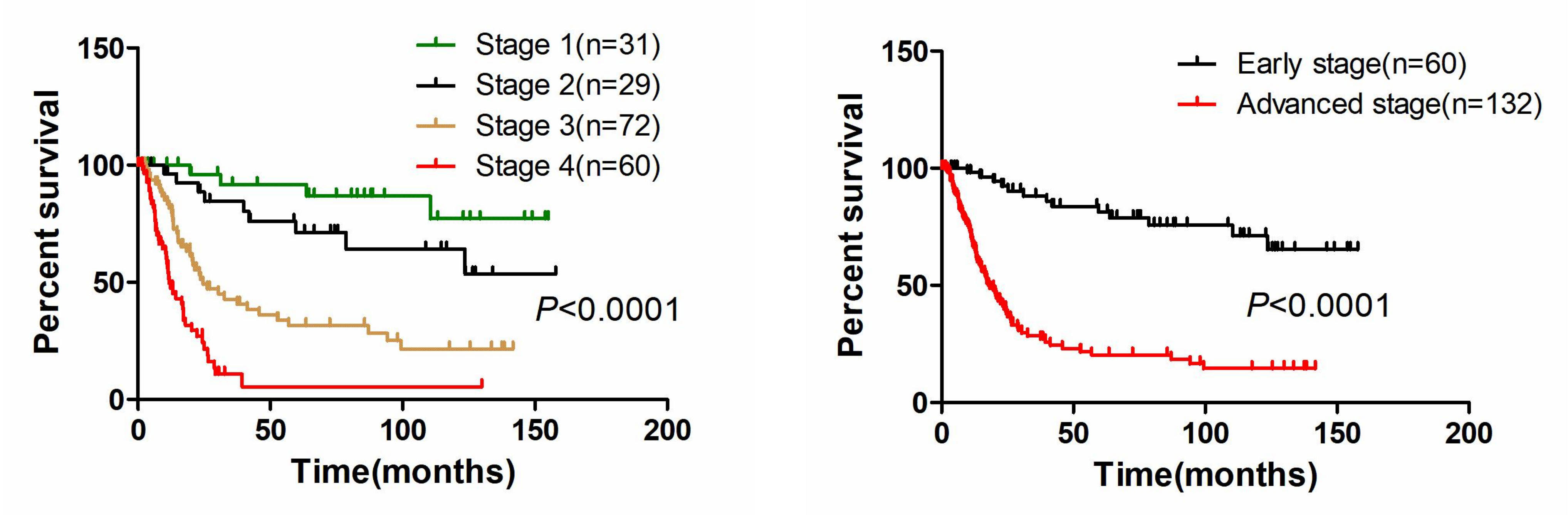

Supplementary Figure 1. Clinical outcomes of patients in different stage of gastric cancer. Early stage included stage 1 and stage 2 patients. Advanced stage included stage 3 and stage 4 patients.

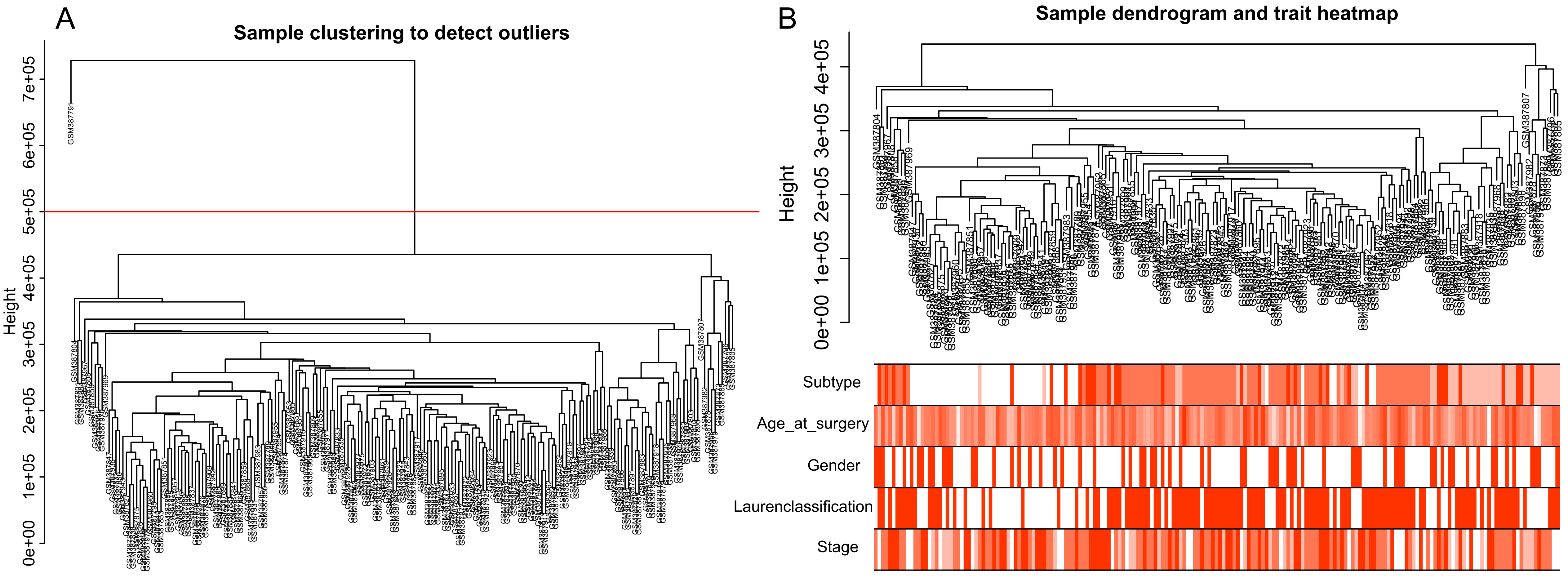

Supplementary Figure 2. Cluster analysis. (A) Cluster analysis of samples to detect outliers. (B) Clustering dendrogram of 191 samples.

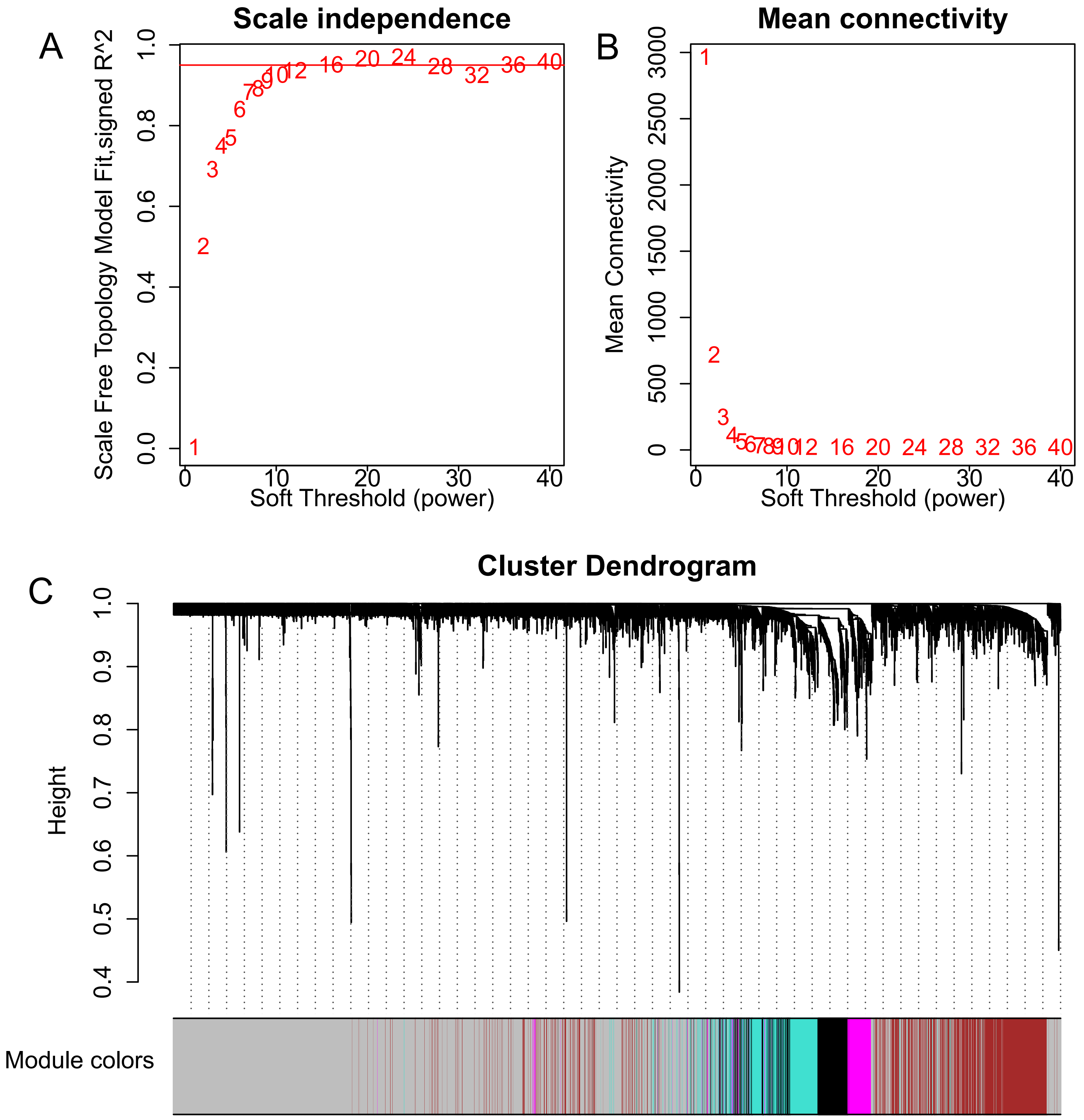

Supplementary Figure 3. Weighted Gene Co-expression Network analysis (WGCNA). (A) The scale independence analysis for various soft threshold powers. (B) The mean connectivity analysis for various soft threshold powers. (C) Clustering dendrogram of genes. Various colors represent different co-expression modules.

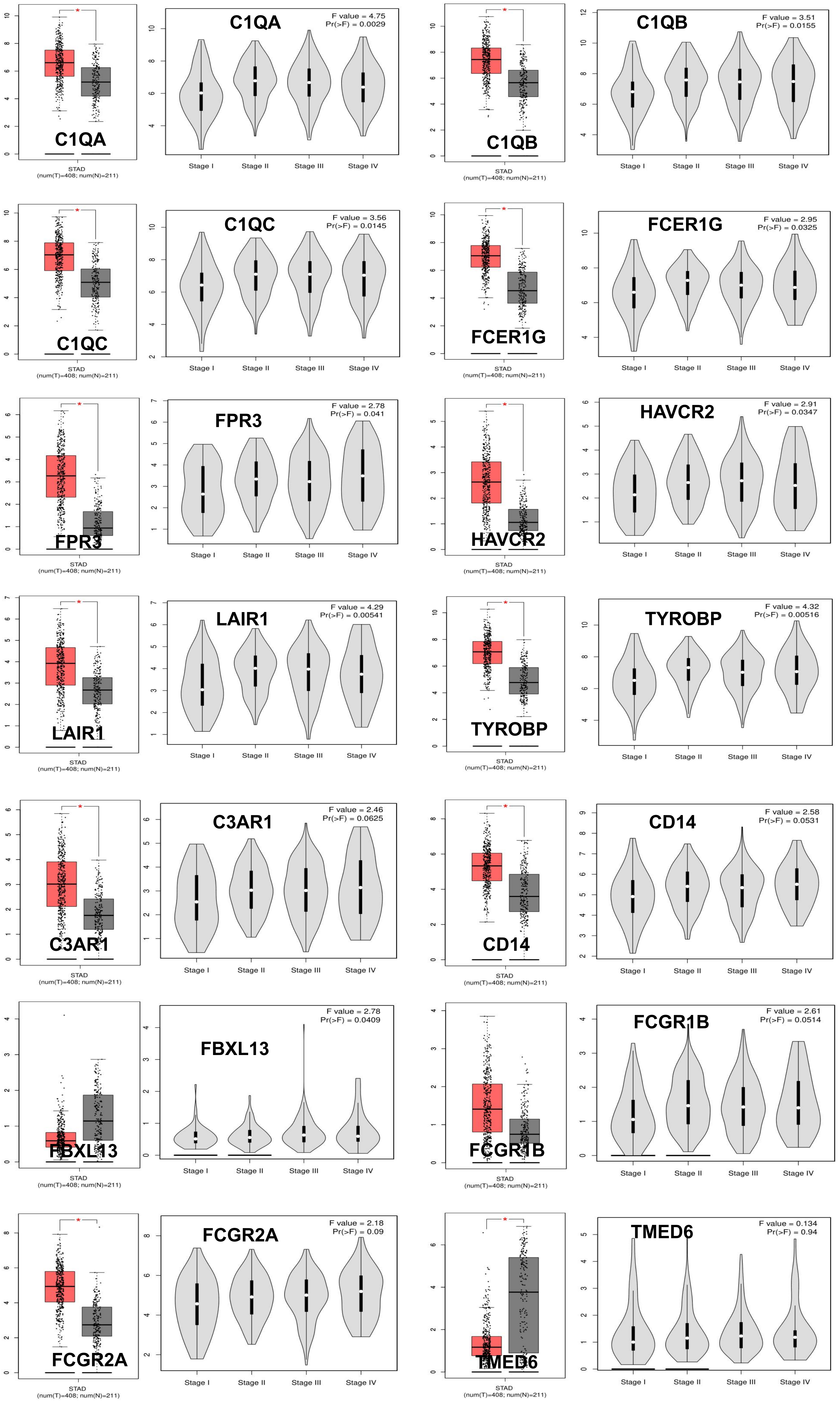

Supplementary Figure 4. The expression of 14 genes in normal tissues and different stage of gastric cancer tissues using The Cancer Genome Atlas (TCGA) database.

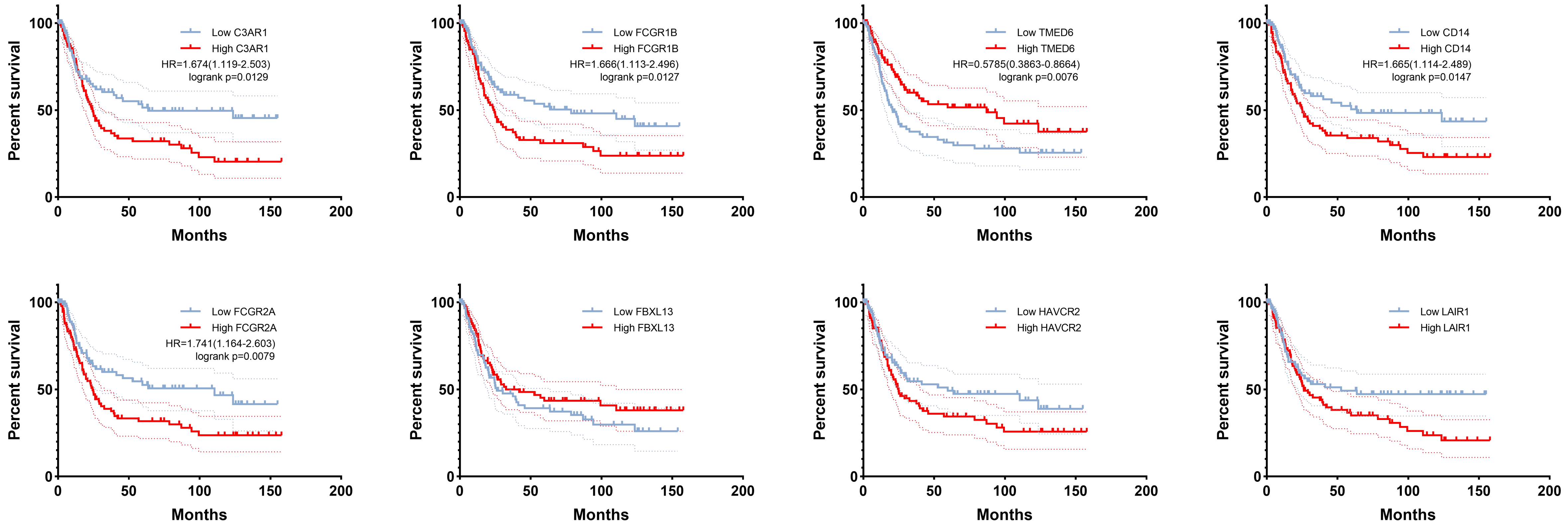

Supplementary Figure 5. The correlation between C3AR1, FCGR1B, TMED6, CD14, FCGR2A, FBXL13, HAVCR2, LAIR1 expression and the prognosis of gastric cancer was analyzed using GSE15459 cohort.
